# Treatment Difficulties in Hypomagnesemia Secondary to the Transient Receptor Potential Melastatin 6 Gene: A Case Report with Novel Mutation

**DOI:** 10.4274/jcrpe.galenos.2020.2020.0004

**Published:** 2021-02-26

**Authors:** Hüsniye Yücel, Çiğdem Genç Sel, Çiğdem Seher Kasapkara, Gülin Karacan Küçükali, Senay Savas-Erdeve, Ülkühan Öztoprak, Serdar Ceylaner, Saliha Şenel, Meltem Akçaboy

**Affiliations:** 1 Dr. Sami Ulus Maternity and Children’s Health and Diseases Training and Research Hospital, Clinic of Pediatrics, Ankara, Turkey; 2 Dr. Sami Ulus Maternity and Children’s Health and Diseases Training and Research Hospital, Clinic of Pediatric Neurology, Ankara, Turkey; 3 Dr. Sami Ulus Maternity and Children’s Health and Diseases Training and Research Hospital, Clinic of Pediatric Metabolism, Ankara, Turkey; 4 Sami Ulus Maternity and Children’s Health and Diseases Training and Research Hospital, Clinic of Pediatric Endocrinology, Ankara, Turkey; 5 Intergen Genetic Center, Ankara, Turkey

**Keywords:** Hypocalcemia, hypomagnesemia, TRPM6, transient receptor potential melastatin 6

## Abstract

Hypomagnesemia is a rare cause of seizures in childhood but should be kept in mind in recurrent and intractable seizures and hypocalcemia in communities where consanguineous marriages are common. Familial hypomagnesemia with secondary hypocalcemia is a rare genetic cause of hypomagnesemia, due to variants in the *transient receptor potential melastatin 6* (*TRPM6*) genes. Here, a three year-old boy with a novel variant in this gene and had difficulties with enteral hypomagnesemia treatment is presented. He had recurrent seizures since two years of age and was diagnosed with epilepsy and treated with multiple antiepileptic drugs. Subsequently, he was diagnosed with rickets due to severe hypocalcemia at another center. The patient was hypotonic and neurodevelopmentally poor. The most prominent laboratory finding was of hypomagnesemia with secondary hypocalcemia. The genetic analysis revealed a novel variant in the TRPM6 gene. After parental treatment of intravenous magnesium (Mg^2+^) sulfate and calcium, the treatment was switched to enteral Mg^2+^ medications, due to persistent hypomagnesemia and the gastrointestinal side-effects, different oral preparations were used. The patient was stable on an oral maintenance dose of Mg^2+^ oxide with borderline blood Mg^2+^ levels and resolution of hypocalcemia. Hypomagnesemia is one of the causes of hypocalcemia. Enteral replacement is the key treatment but the treatment should be individualized for each patient. Normalization of hypomagnesemia is not always easy and should not be the aim of the treatment.

**What is already known on this topic?**

Hypomagnesemia is one of the causes of hypocalcemia. Enteral replacement is the key treatment but the treatment should be individualized for each patient. Normalization of hypomagnesemia is not always easy and should not be the aim of the treatment.

**What this study adds?**

Genetic analysis revealed a novel frame shift variant in the transient receptor potential melastatin 6 gene. Magnesium levels varied during treatment with different preparations so in these patients treatment should be individualized for optimal replacement.

## Introduction

Familial hypomagnesemia with secondary hypocalcemia (HSH) is a rare autosomal recessive disorder that presents in infancy with neurological symptoms of magnesium (Mg^2+^) dependent hypocalcemia (1,2). Variants in the gene for the distal convoluting tubules and colon specific apical Mg^2+^ channel, the transient receptor potential melastatin 6 (*TRPM6*) gene, cause the most profound genetic hypomagnesemia (3). To date, there are a few reports of several variants of *TRPM6* in children (4). In addition, some reports have referred to the difficulties of maintaining serum Mg^2+^ levels in these patients (3,4). The treatment complexities, including the target serum Mg^2+^ levels and the different options for preparations to be given were rarely reported. Therefore, we present a patient with resistant seizures who was diagnosed with HSH due to a novel variant in *TRPM6* gene, discuss the importance of checking Mg^2+^ in seizures and to consider and discuss treatment strategies.

## Case Report

**Figure 1:**
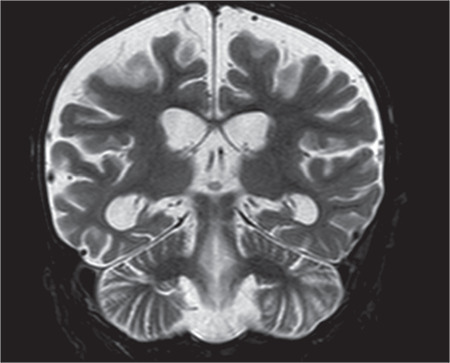
Cerebral and cerebellar atrophy in T2-weighted cranial magnetic resonance images

A three-year-old Afghan boy was admitted to our hospital with a history of recurrent seizures since the age of two years. The first seizure was reported to be fever-induced at the age of four months. Cognitive and motor development was normal until the age of one, but thereafter neurodevelopmental decline was reported. He had been having generalized tonic-clonic seizures since the age of two years. The diagnosis of epilepsy was made in another center because of recurrent seizures and multiple antiepileptic drugs were started. He has been treated with levetiracetam, clonazepam, valproic acid, and pyridoxine treatments. The patient was admitted to our hospital from Afghanistan for further evaluation for recurrent seizures and hypocalcemia. He was born at full-term gestation after an uncomplicated pregnancy including an absence of polyhydramnios with a birth weight of 3500 gr from a consanguineous family (first degree cousins). His prenatal and natal history was uneventful. His family history was unremarkable. His family history was negative for epilepsy and neurological abnormalities, as well as any known renal, thyroid, or parathyroid disease. Physical examination revealed his growth parameters were within normal limits [height: 95 cm (25-50 p), weight: 15 kg (50-75 p)]. He did not show any dysmorphic or neurocutaneous features. He was conscious and had a speech delay. Bilateral horizontal nystagmus was prominent. He was hypotonic with normal reflexes. The rest of the physical examination was normal. Laboratory data included a serum Mg^2+^ level of 0.12 mmol/L (normal range: 0.7-0.86 mmol/L), calcium 4.7 mg/dL, phosphorus 6.2 mg/dL, alkaline phosphatase 222 U/L, parathyroid hormone 6 pg/mL (normal range: 11-67 pg/mL), 25-OH vitamin D3 60.1 ng/mL, sodium 139 mEq/L, potassium 4.24 mEq/L, albumin 3.2 g/dL, and uric acid 5.3 mg/dL. The urine fractional excretion of Mg^2+^ was 12% (normal range: <4%) with normal urine calcium/creatinine ratio. Laboratory examinations including acute phase reactants, serum glucose, and albumin levels, and liver and renal function tests were normal. The renal ultrasound did not show any medullary nephrocalcinosis. Electroencephalogram showed slow background activity without any epileptiform discharges and magnetic resonance imaging of brain showed mild diffuse cerebral and cerebellar atrophy (Figure 1).

**Figure 2:**
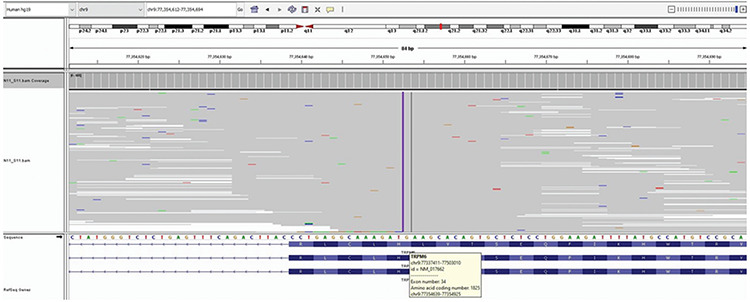
The figure of the pathogenic variant in the transient receptor potential melastatin 6 genes

Laboratory examination revealed the characteristic combination of severe hypomagnesemia, hypoparathyroidism, and profound hypocalcemia. Clinical and laboratory findings together suggested the diagnosis of HSH as responsible for disruption in Mg^2+^ homeostasis. The genetic analysis of the patient revealed a novel, homozygous variant in the *TRPM6* gene (NM017662.4: c.5473_5474insGCTTC (p.H18225Rfs*18) (p.His1825Argfs*18). This is a frameshift variant and based on American College of Medical Genetics and Genomics criteria this variant was classified as pathogenic. This is a null variant and is predicted to cause severe loss of gene function. *TRPM6* gene sequence analysis was performed using MiSeq next generation sequencing platform, an Food and Drug Administration approved diagnostic system (Illumina Inc., San Diego, CA, USA). Sequences were aligned to the hg 19 genome within MiSeq Reporter software (Illumina Inc.). Visualization of the data was performed with IGV 2.3 (Broad Institute-www.software.broadinstitute.org) software (Figure 2). Informed consent was obtained from the parents for genetic analysis. The parents were heterozygotes for the same variant.

**Figure 3:**
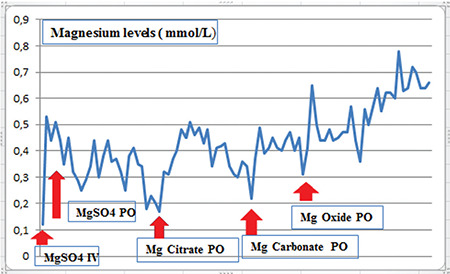
The blood magnesium levels by different magnesium preparations Mg: magnesium, MgSO4: magnesium sulphate, IV: intravenous; PO: per oral

Intravenous Mg^2+^ sulfate was administered at 50 mg/kg along with intravenous calcium for three days after which the serum Mg^2+^ increased and calcium levels normalized. The treatment was switched to oral Mg^2+^ sulfate 4x2000 mg (~533 mg/kg/d) but abdominal pain and diarrhea was significant. In addition, on this oral therapy, serum Mg^2+^ decreased to 0.2 mmol/L and convulsions re-occurred without hypocalcemia. The treatment was replaced with oral Mg^2+^ citrate and soon after switched again to Mg^2+^ carbonate because of persistent hypomagnesemia and gastrointestinal side-effects. Finally, Mg^2+^ oxide sachets were started and blood Mg^2+^ reached 0.78 mmol/L with high dose Mg^2+^ oxide (3x2 sachets ~ 150 mg/kg/d) (Figure 3). Antiepileptic treatment was reduced. His muscle tone, cognitive development, and motor development improved. He has been stable on an oral maintenance dose of 2000 mg of Mg^2+^ oxide daily with borderline blood Mg^2+^ levels without hypocalcemia.

## Discussion

We presented a case of HSH due to a novel variant in the *TRPM6* gene. The patient’s treatment was individually tailored according to blood Mg^2+^ levels and to minimize the side effects of a range of Mg^2+^-containing medications. The patient benefitted from Mg^2+^ replacement for neurodevelopmental improvement and showed satisfactory progress.

HSH is a rare autosomal recessive disorder that affects the Mg^2+^ permeable ion channel encoded for by *TRPM6* gene on chromosome 9q22 (3). This gene is expressed in the distal segment of the intestine and the distal convoluted renal tubule. So the primary defect is impaired intestinal absorption of Mg^2+^ with a secondary defect of impaired renal conservation. The clinical presentation is usually in the early childhood period with hypocalcemia refractory to calcium supplementation. This secondary hypocalcemia is probably caused by inhibition of the parathyroid gland by the hypomagnesemia, resulting in low levels of parathyroid hormone which eventually results in hypocalcemia (1,4,5). The condition is treatable, but failure to diagnose early can lead to intractable seizures with irreversible cerebral damage and mental retardation (1). Some reports have revealed initial evaluations for neonates and infants presenting with seizures do not always include assessment for serum Mg^2+^ abnormalities (3,6). As far as the treatment is dependent on lifelong, high-dose supplementation of Mg^2+^ and the genetic diagnosis is relevant, this disorder should be included in the differential diagnosis of any infant presenting with seizures and hypomagnesemia. Our patient was being followed for intractable epilepsy as well as rickets for two years. Neurodevelopmental delay and recurrent seizures increased the suspicion of neurometabolic disorders with the family history of consanguinity. The need to check Mg^2+^ levels in a severely hypocalcemic patient was overlooked.

Previous reports of HSH have demonstrated how well-timed diagnosis and rapid Mg^2+^ replacement accelerate normal development (3). One case series described considerably impaired neurodevelopment in two affected members of the same family who failed to receive supplementation (7). In another report, a patient who had HSH due to a *TRPM6* variant was followed-up over 29 years and demonstrated normal physical and mental development with treatment (3). The reported patient showed normal developmental milestones, she completed her education including getting a university science degree and went on to follow an academic career as an adult. The diagnosis age of the patients in the literature ranges from the neonatal period to four years old. The neurological outcome is reported to be related to the age at diagnosis and also the compliance to the treatment (5). Hypomagnesemia itself leads to lethargy, nystagmus and convulsions. In addition, without suitable treatment, it can lead to cerebral atrophy as was found in the present case (8,9). Even short-term follow-up of our patient demonstrated neurodevelopmental progress in our patient with appropriate treatment.

Oral or intravenous Mg^2+^ supplementation is the only existing treatment for hypomagnesemia of genetic origin. In the acute symptomatic situation of severely hypomagnesemic patient, intravenous Mg^2+^ supplementation is critical (1). The optimal rise in serum Mg^2+^ concentration often improves symptoms, such as seizures and secondary hypocalcemia, despite the fact that normal blood Mg^2+^ values are rarely reached (2,10). Extended correction of hypomagnesemia is generally delayed because of the gastro-intestinal side effects frequently associated with oral Mg^2+^ supplementation. Paradoxically, higher doses of oral Mg^2+^ is damaging to intestines and results in diarrhea and also worsening hypomagnesemia (1,11). Thus, the type of oral Mg^2+^ preparation is important, since some preparations have been reported to have a better bioavailability than others (1,10). In a recent report, Mg^2+^ chloride or Mg^2+^ glycerophosphate was suggested rather than Mg^2+^ oxide or Mg^2+^ sulfate for oral Mg^2+^ supplementation (1). In a study conducted in mice, different Mg^2+^ preparations (Mg^2+^ acetyl taurate; Mg^2+^ malate; Mg^2+^ glycinate; Mg^2+^ citrate) were shown to be effective in increasing Mg^2+^ levels in different tissues like brain and muscle (12). In that study, blood Mg^2+^ levels were increased in all doses of Mg^2+^ acetyl taurate, malate, and glycinate, whereas Mg^2+^ citrate increased blood Mg^2+^ levels at high doses. Mg^2+^ citrate was reported to lead to a dose dependent increase in blood, brain, and muscle tissues (12). However, in our patient the highest serum Mg^2+^ levels without apparent side effects was achieved with Mg^2+^ oxide supplementation. This highlights the importance of tailoring the treatment to the individual patient as acceptability of Mg^2+^ supplement treatments vary from patient to patient. Treatment options may be optimized, based on future studies that investigate tissue-dependent Mg^2+^ concentrations in humans.

Milder clinical phenotypes may be due to different variants. However, due to the rarity of the condition, no definitive genotype–phenotype correlation has been established at present. The most commonly reported symptoms on admission were recurrent and intractable myoclonic or generalized tonic-clonic seizures (4). Mg^2+^ transport in the intestine occurs by both an active transcellular system, which is defective in HSH, and a passive paracellular pathway, which increases with rising intraluminal Mg^2+^ concentrations (1,2,3,4). Therefore, lifelong, enteral, high-dose Mg^2+^ is required in HSH to prevent symptoms and achieve at least subnormal serum Mg^2+^ levels. Optimal doses have been identified by trial and error and serial serum electrolyte monitoring. Previously reported cases have shown serum Mg^2+^ levels remain in the subnormal range (0.5-0.6 mmol/L) even with significant increases in supplemented dose (3). The published data suggests that the clinical aim should be normocalcaemia and the absence of features of neuroexcitability (3,8). There is no preparation of choice for oral Mg^2+^ replacement and preparation selection should be guided by follow-up in each individual patient and may also be affected by differences in the specific variant inherited.

*TRPM6* variant is a cause of profound hypomagnesemia with secondary hypocalcemia. With appropriate treatment, the seizures can be controlled and neurocognitive development of the patients can be improved. Rapid diagnosis and treatment of this rare disorder can significantly improve the quality of life of affected individuals.

Hypomagnesemia is one of the causes of hypocalcemia. A diagnosis of primary HSH should be considered in all pediatric patients presenting with generalized seizures or tetany. Measurement of serum Mg^2+^ should be included in the work-up, especially during a seizure episode. This is especially true in those communities where consanguineous marriages are common. Enteral or parenteral Mg^2+^ replacement is key in managing this condition and the aim should be to normalize serum calcium and control the symptoms. The treatment of hypomagnesemia is not always easy and may depend on the dose and the content of the medication. Individualized therapy and management should be tailored to each patient.
